# Simvastatin Upregulates Lipoxin A4 and Accelerates Neuroinflammation Resolution After Intracerebral Hemorrhage

**DOI:** 10.2174/1567202619666220913124627

**Published:** 2022-12-27

**Authors:** Jianbo Zhang, Na Hao, Wei Li, Qianwei Chen, Zhi Chen, Hua Feng, Yao Wu, Xia Shi

**Affiliations:** 1 Department of Neurosurgery, General Hospital of South Theater Command, Guangdong, 510010, China;; 2 Department of Neurosurgery, Southwest Hospital, Third Military Medical University (Army Medical University), Chongqing, 400038, China;; 3 Department of Orthopedics, Chongqing Hospital of Traditional Chinese Medicine, Chongqing, 400000, China;; 4 Department of Laboratory Medicine, Western Theater Command Air Force Hospital, Chengdu, 610065, China;; 5 Department of Neurosurgery, Chongqing University Three Gorges Hospital, Chongqing, 404031, China;; 6 Department of Nutrition, Southwest Hospital, Third Military Medical University (Army Medical University), Chongqing, 400038, China

**Keywords:** Intracerebral hemorrhage, statins, neutrophils, apoptosis, lipoxin A4, formyl-peptide receptor 2, inflammation

## Abstract

**Background:**

Previous studies have demonstrated that statins can relieve inflammatory brain injury after intracerebral hemorrhage (ICH), but the mechanisms remain poorly characterized. This study aims to test whether simvastatin exerts an anti-inflammatory effect by regulating the pro-resolving mediators.

**Methods:**

First, male Sprague–Dawley rats had an injection of 200 μL autologous blood. Then, rats were randomly divided into groups treated with simvastatin (i.p. 2 mg/kg) or vehicle. Next, all rats underwent pro-resolving mediator lipoxin A4 (LXA4) level detection, flow cytometric, immunofluorescence, brain edema measurement, neurological scoring and western blot analysis.

**Results:**

We found that simvastatin significantly increased the plasma level of LXA4, an endogenous formyl-peptide receptor 2 (FPR2) agonist, in the early stage of ICH. Consistent with the effect of simvastatin, exogenous LXA4 administration also promoted apoptosis of the circulating neutrophils, reduced neutrophils brain infiltration, and ameliorated inflammatory brain injury after ICH. In addition, similar to simvastatin, exogenous LXA4 markedly decreased the level of phosphorylated p38 mitogen-activated protein kinase (MAPK) and the apoptosis-related proteins myeloid cell leukemia 1(Mcl-1)/Bax ratio (a decreased ratio represents the induction of apoptosis) in circulating neutrophils isolated from ICH rats. Notably, all of the aforementioned effects of simvastatin on ICH were significantly abolished by Boc-2, a selective antagonist of FPR2. Moreover, simvastatin led to a similar Mcl-1/Bax ratio reduction as SB203580 (a p38 MAPK inhibitor), but it was abolished by P79350 (a p38 MAPK agonist).

**Conclusion:**

Collectively, these results suggest that simvastatin ameliorates ICH-mediated inflammatory brain injury, possibly by upregulating the level of pro-resolving mediator LXA4 and further stimulating the FPR2/p38 MAPK signaling pathway.

## 1 INTRODUCTION

Intracerebral hemorrhage (ICH) is a life-threatening illness of global importance, with a poor prognosis and few proven treatments. One in three patients dies within the first month of onset. Survivors have varying degrees of residual disability and a high risk of recurrent ICH [1, 2]. An increasing number of studies have provided evidence supporting the key role of neuroinflammation in secondary brain injury following ICH [3].

Statins, a classic cholesterol-lowering agent, have been widely used to fight cardiovascular diseases and ischemic stroke [4]. In addition to cholesterol control, statins also exhibited beneficial pleiotropic effects, such as anti-inflammation, preventing superoxide free radicals and thrombus formation, and improving angiogenesis, synaptogenesis and neurogenesis [5]. Many retrospective studies [6-10] found a link between statin use, lower mortality, and better outcomes in ICH patients. However, no authoritative prospective randomized trials have verified these effects so far.

Recently, clinical reports have demonstrated that an early increase in peripheral polymorphonuclear neutrophil (PMN) count is closely related to a poor prognosis of patients with ICH, which is often accompanied by increased brain edema and larger hematoma [11-14]. Therefore, to explore the potential anti-inflammation effect of stains for ICH, we performed a confirmatory experiment in a rat model of ICH [15]. The ICH rats showed a marked PMN count increase in peripheral blood at an early stage, which is consistent with the clinical phenomenon. Interestingly, the increased PMN count returned to normal in animals pretreated with simvastatin. In addition, neutrophil brain infiltration, neuroinflammation, brain edema and neurological dysfunction in ICH rats were significantly ameliorated after simvastatin use. However, to date, the related molecular mechanisms are not clear.

Lipoxin A4 (LXA4) is an important endogenous lipid synthesized by 5-lipoxygenase and exerts potent anti-inflammatory effects by inhibiting PMN infiltration and pro-inflammatory cytokine release [16]. In recent years, studies have reported that LXA4 exerts a notable anti-inflammatory and neuroprotective effect by activating its receptor formyl peptide receptor 2 (FPR2) in animal models of ischemic stroke, subarachnoid hemorrhage and ICH [17-19]. LXA4 exerted these biological functions by downregulating p38 mitogen-activated protein kinase (MAPK), which was mediated by FPR2 [18, 20]. Recently, González-Herrera *et al.* [21] found that simvastatin assists cyclooxygenase-2 (COX-2) in inducing the conversion of arachidonic acid to LXA4 and exerts anti-inflammatory effects. Furthermore, El Kebir *et al.* [22] discovered that LXA4 effectively promotes PMN apoptosis and reduces subsequent PMN infiltration throughout the respiratory tract by accelerating the degradation of myeloid cell leukemia 1 (Mcl-1), a key anti-apoptotic protein. Therefore, based on these findings, we speculate that simvastatin may exert an anti-inflammatory effect following ICH by upregulating the endogenous pro-resolving mediators LXA4.

## 2 MATERIALS AND METHODS

### 2.1 Animals and the ICH Model

The Institutional Animal Care and Use Committee at the Army Medical University approved this study (SCXK-PLA-20120011), and the procedures were performed in accordance with institutional guidelines. Two hundred eighty-five adult male Sprague–Dawley rats (250-350 g) were used. The ICH model was established as described in our previous studies [5, 23]. Briefly, non-anticoagulated whole blood was collected from the femoral artery of rats. Then, autologous blood was microinjected into the right caudate nucleus within 10 min. The coordinates were 0.2 mm anterior, 5.5 mm ventral, and 3.5 mm lateral to the bregma. The sham groups received only a needle injection.

### 2.2 Experimental Protocol

#### 2.2.1 The Present Study Conducted Three Separate Experiments, as Described Below

##### 2.2.1.1 Experiment 1

Twenty-four rats were randomly divided into three groups to determine simvastatin-induced changes in plasma LXA4 levels after ICH: sham, ICH + Veh. (ICH + saline), and ICH + Simva. (ICH + simvastatin). Plasma levels of LXA4 were assessed using enzyme-linked immunosorbent assays (ELISAs) at 24 h and 72 h following blood injection (n = 4 rats per group at each time point).

##### 2.2.1.2 Experiment 2

We randomly assigned 141 rats into the following five groups to validate the involvement of the LXA4/FPR2 pathway in simvastatin-mediated induction of peripheral PMN apoptosis and the subsequent alleviation of neuroinflammation after ICH: Sham, ICH + Veh, ICH + LXA4 (an endogenous FPR2 agonist), ICH + Simva., and ICH + Simva. + Boc-2 (a selective FPR2 antagonist). Because our previous study provided some corresponding data for the sham group, the sham group underwent measurements of PMN counts and pro-inflammatory factor protein levels in the present experiment. Flow cytometry analysis of peripheral PMN apoptosis (n = 6 rats per group), routine blood counts and immunofluorescence staining for myeloperoxidase (MPO) (n = 6 rats per group), and Western blot analysis of pro-inflammatory factors (n = 3 rats per group) were conducted 24 h after ICH induction. The brain water content measurement (n = 6 rats per group), modified neurological severity scores (mNSS, n = 6 rats per group) and corner tests (n = 6 rats per group) were assessed at 1, 3 and/or 7 days after ICH.

##### 2.2.1.3 Experiment 3

Levels of p38 MAPK, the potential downstream signaling pathway of FPR2-mediated PMN apoptosis, were evaluated in PMNs isolated from the circulating blood of ICH rats. First, 12 rats were randomly divided into four groups: ICH + Veh., ICH + LXA4 (an endogenous FPR2 agonist), ICH + Simva., and ICH + Simva. + Boc-2 (an FPR2 selective antagonist). The Western blot analyses of p38, pp38, Mcl-1, Bax levels and the Mcl-1/Bax ratio (n = 3 rats per group) were conducted 24 h after ICH. Next, another 12 rats were randomly divided into the following four groups: ICH + Veh., ICH + SB203580 (p38 MAPK pathway inhibitor), ICH + Simva., and ICH + Simva. + P79350 (p38 MAPK pathway agonist). 24 h after ICH, Western blot analyses of Mcl-1 and Bax levels and the Mcl-1/Bax ratio (n = 3 rats per group) were conducted.

### 2.3 Drug Administration

First, simvastatin (Sigma, United States) was prepared as a 4 mg/ml solution, as described in our previous study [15]. This simvastatin stock was stored at 80°C and immediately diluted with a triple volume of sterile saline before use. Animals received a simvastatin injection (2 mg/kg/d, i.p.) beginning five days before ICH until sacrifice. The FPR2 antagonist Boc-2 (100ug/kg, Nanjing Peptide Industry, CHN) or exogenous LXA4 reagent (10ug/kg, Cayman Chemical, USA) was injected through the abdominal cavity 30 min before ICH [18].

### 2.4 Detection of LXA4 Levels

3 ml of circulating blood was collected from the rat heart and stored in an EDTA-anticoagulant tube 24 or 72 hours after ICH. The upper yellow plasma layer was removed and stored in a refrigerator at -80°C for later use. The LXA4 level was measured using the rat LXA4 ELISA kit (Shanghai Jianglai Biological Co., LTD, CHN) according to the manufacturer’s instructions.

### 2.5 Routine Blood Counts

Routine blood counts were conducted as previously described [15]. First, 4 ml of circulating blood was collected from the rat heart in an EDTA-anticoagulant tube. Then, the tube was shaken vigorously. Next, 200 µL of the sample was transferred to an Eppendorf tube and tested with a bench-top analyzer (Hemavet 950, Shandong Excellent Science Instrument Co. LTD., CHN).

### 2.6 PMN Isolation

Our previous study [15] described that the anticoagulant-treated whole blood was collected from rats and subjected to density gradient centrifugation. Then, PMN cells were collected from the PMN-rich layer between Histopaque1083 and Histopaque1119. Next, cell viability was examined with Trypan blue dye. The purity of isolated PMNs was tested with Wright-Giemsa staining. These isolated PMNs exhibited greater than 95% viability and purity.

### 2.7 Determination of the Ratio of Apoptotic PMNs

The ratio of apoptotic PMNs was detected using a previously reported method [15]. Cells were washed with D-Hanks buffer and incubated on ice with 10 μL of propidium iodide (PI) solution and 5 μL of Annexin V-fluorescein isothiocyanate (FITC) solution for 15 min in the dark. Then, the apoptosis of PMNs was analyzed using flow cytometry (BD LSRF Ortessa, USA).

### 2.8 Brain Water Content Measurement

As previously described [15], brain water content was examined in rats at 24 h and 72 h after surgery. Rat brains were removed under deep anesthesia, and the tissue was sliced into coronal sections (4 mm thickness) around the injection needle tract. Brain sections were divided into four parts: ipsilateral basal ganglia, ipsilateral cortex, contralateral basal ganglia, and contralateral cortex. The cerebellum serves as the internal control. Brain samples were immediately weighed after removal and then dried for 24 h in a 100 °C oven. The brain water content (%) was calculated as (wet weight-dry weight)/wet weight × 100%.

### 2.9 Assessments of Neurological Function

Neurological assessments were conducted with the mNSS method and the corner test, as previously described [24, 25]. In brief, the mNSS scale ranges from 0 to 18 points (normal score, 0; maximal deficit score, 18). In the corner test, each rat was placed in a corner at a 30° angle. Alternatively, the rat was allowed to turn left or right. After 10 repeated tests, the ratio of right turns was calculated by two blinded observers.

### 2.10 Cell Counts

Cells were counted in brain sections. Three high-power images were captured around the hematoma using a confocal microscope (LSM-780; Zeiss). 1, 3, and 7 days after ICH, MPO-positive cells were counted by two researchers in a blinded manner. The counts were performed on four consecutive brain sections.

### 2.11 Western Blot Analysis

Cell lysates were separated on SDS–PAGE gels, and proteins were detected on nitrocellulose blots with enhanced chemiluminescence reagents (GE Healthcare). The following antibodies were used: TNF-α, diluted 1:500 (Beyotime, China); IL-6, diluted 1:500 (Beyotime, China); Mcl-1, p38 MAPK and phospho-p38 MAPK (1:1000, Cell Signaling, USA); Bax (1:500, Abcam, USA); complement C3 (1:500, Novus Biologicals, USA); α-tubulin (1:1000, Boster Biological Technology, China); and GAPDH (1:1000, bs-2188R; BIOSS, China). Membranes were blocked with 5% nonfat milk dissolved in Tris-buffered saline solution for 1 h, followed by overnight incubation with the primary antibody [26]. Detailed information about antibodies is shown in the attached list (Table **1**).

### 2.12 Immunofluorescence Staining

As previously described [15], the brain tissue immunofluorescence staining was performed on fixed frozen sections. Serial sections were cut using a freezing microtome, blocked, and incubated with the primary antibody against MPO (1:100, Abcam, USA). After washing, the sections were incubated for 3 hours at 37°C with Alexa Fluor 555-conjugated goat anti-rabbit IgG (H+L) (1:300, Beyotime, China) secondary antibody counterstained with DAPI.

### 2.13 Statistical Analysis

In the present study, the values are reported as the means±SD. Data were analyzed using a one-way analysis of variance, followed by Scheffe’s post hoc test. Differences were considered statistically significant at a *P* value < 0.05.

## 3 RESULTS

### 3.1 Simvastatin Significantly Increased the Plasma Level of Pro-resolving Mediators like LXA4 (an Endogenous FPR2 Agonist) at 24 h After ICH

We selected LXA4, an endogenous FPR2 agonist, and detected its levels to examine the potential correlation between simvastatin and FPR2 after ICH. An ELISA was performed to determine the profile of LXA4 expression at 24 h and 72 h after ICH. As shown in Fig. (**1**), compared with the sham and control groups, the plasma of ICH rats treated with simvastatin contained higher levels of LXA4 at 24 h after blood injection but showed no significant difference at 72 h. This result suggests that simvastatin has the potential to regulate FPR2 by altering the LXA4 level.

### 3.2 Boc-2, a Selective FPR2 Antagonist, Reduced Simvastatin-Mediated Peripheral PMN Apoptosis

**Fig. (2):**
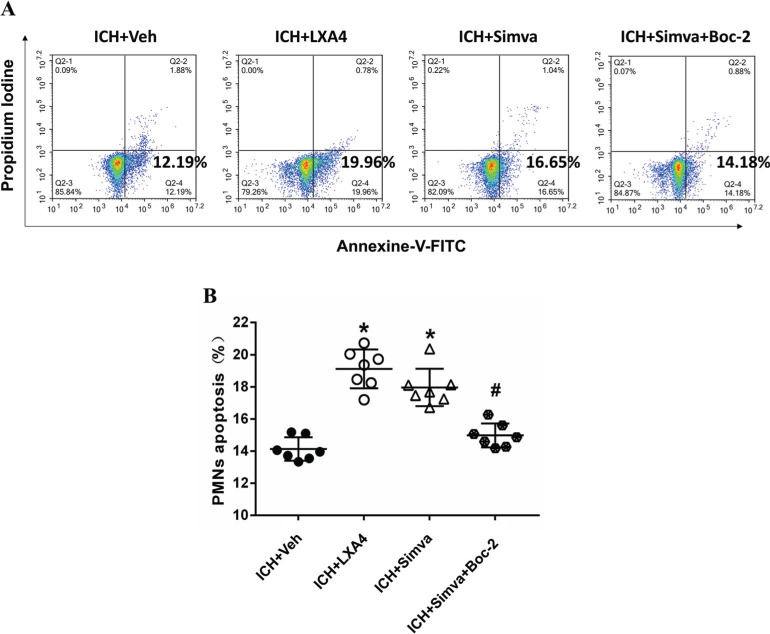
**Boc-2, a selective FPR2 antagonist, reduced simvastatin-mediated peripheral PMN apoptosis.** To determine the role of FPR2 in simvastatin-induced apoptosis of PMN, we conducted a flow cytometry analysis at 24 h after ICH. Compared with the control group, both the LXA4 and Simva-groups showed a higher apoptotic ratio (**A** and **B**). The LXA4 group displayed a more prominent increase. Notably, Boc-2, a selective FPR2 antagonist, counteracted simvastatin-induced PMN apoptosis (**A** and **B**), suggesting FPR2 was involved in simvastatin/LXA4-triggered PMN apoptosis. The apoptotic ratio was calculated from the ratio of apoptotic cells to total cells counted. Values are presented as means±SD, n=7 rats per group; *P<0.05 compared with the ICH+Veh group; #*P<*0.05 compared with the ICH+Simva group. PMN stands for polymorphonuclear neutrophil.

We conducted a flow cytometry analysis using exogenous LXA4 (an FPR2 agonist) and Boc-2 (an FPR2 antagonist) at 24 h after ICH to determine the role of FPR2 in simvastatin-induced apoptosis. Compared with the control group, the LXA4 and Simva-groups displayed a higher ratio of apoptotic PMNs (Figs. **2A** and **B**). The LXA4 group showed a more prominent increase. Notably, Boc-2, a selective FPR2 antagonist, markedly reduced simvastatin-induced PMN apoptosis (Figs. **2A** and **B**).

### 3.3 Boc-2 Reversed the Inhibitory Effects of Simvastatin on the PMN Count and PMN Invasion in the Brain

**Fig. (3):**
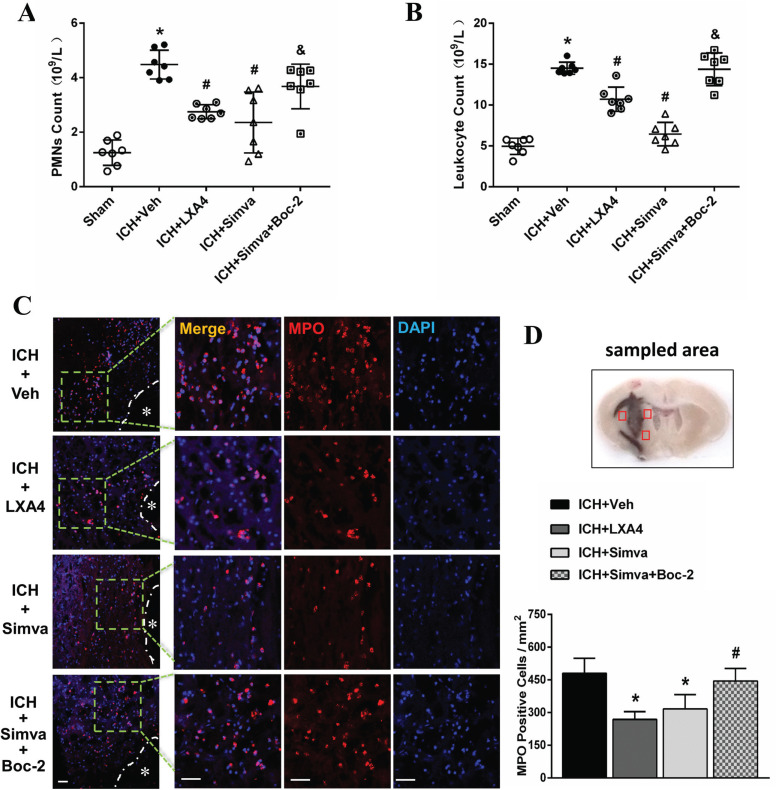
**Boc-2, a selective FPR2 antagonist, blocked the inhibitory effects of simvastatin on peripheral PMN counts and PMN invasion in the brain. (A** and **B**) Routine blood tests showed that ICH rats treated with the exogenous LXA4 (an FPR2 agonist) had lower PMNs and leukocyte count than controls 24 h post-ICH. Interestingly, the simvastatin-treated rats showed a similar trend to the LXA4 group, but Boc-2 reversed the inhibitory effect of simvastatin. Values are presented as the means±SD, n=7 rats per group; *P<0.05 with the sham group; #*P<*0.05 with the Veh group; & *P<*0.05 with the Simva-group. (**C** and **D**) Immunofluorescence assay showed that ICH rats treated with LXA4 performed less PMN infiltration into the brain than the control 24 h post-ICH. Notably, the Simva-group presented a similar trend to the LXA4 group, but Boc-2 also abolished the inhibitory effect of simvastatin on PMN invasion. Values are presented as means±SD, n=6 rats per group; *P<0.05 with the Veh-group; #*P<*0.05 with the Simva-group.

Next, we investigated the role of FPR2 in the effects of simvastatin on modulating circulating PMN counts and PMN infiltration into the brain after ICH. Routine blood tests showed that ICH rats treated with the FPR2 agonist LXA4 had a lower PMN count than the control rats at 24 h after ICH (Figs. **3A** and **B**). Interestingly, the simvastatin-treated animals showed a similar trend to the LXA4 group, but the inhibitory effects of simvastatin on PMN counts were balanced by the FPR2 antagonist Boc-2 (Figs. **3A** and **B**). As displayed in Figs. (**3C** and **D**), compared with the non-treated ICH group, exogenous LXA4 (an FPR2 agonist) administration significantly decreased the number of MPO (+) cells in the area around the hematoma at 24 h post-ICH. Notably, the Simva-group presented a similar trend to the LXA4 group, but the inhibitory effects of simvastatin on PMN invasion into the brain were reversed by Boc-2 (FPR2 antagonist).

### 3.4 The Anti-inflammatory Effect of Simvastatin on ICH was Abrogated by Boc-2

**Fig. (4):**
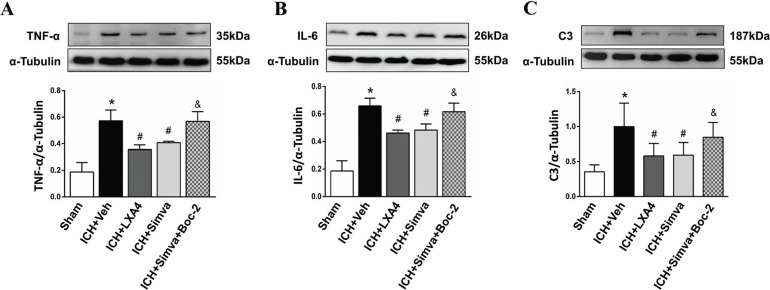
**Boc-2, a selective FPR2 antagonist, counteracted the anti-inflammatory effect of simvastatin on ICH rats.** TNF-α (**A**), IL-6 (**B**), and complement component C3 (**C**) protein levels were lower in the LXA4-group than in the control group 24 hours after ICH, indicating that FPR2 stimulation reduced the early inflammatory response to ICH. In particular, the Simva-group exhibited a similar trend to the LXA4 group, but Boc-2 neutralized the anti-inflammatory effect of simvastatin on ICH. Values are presented as the means ± SD, n=3 rats per group; *P<0.05 with the Sham group; #*P<*0.05 with the Veh-group; & *P<*0.05 with the Simva-group.

Additionally, we determined changes in the levels of some pro-inflammatory factors around the hematoma by detecting the expression of the TNF-α, IL-6 and complement component C3 proteins in the acute stage of ICH. At 24 h after ICH, Western blot analyses showed lower expression levels of all of these chemokines in the LXA4 group than in the control group (Figs. **4A**-**C**), indicating that FPR2 stimulation relieved the early neuroinflammatory response to ICH to some extent. In particular, the Simva-group exhibited a similar trend to the LXA4 group, but Boc-2, a selective FPR2 antagonist, neutralized the anti-inflammatory effect of simvastatin on ICH.

### 3.5 The Protective effects of Simvastatin on Brain Edema and Neurological Deficits Were Abolished by Boc-2

**Fig. (5):**
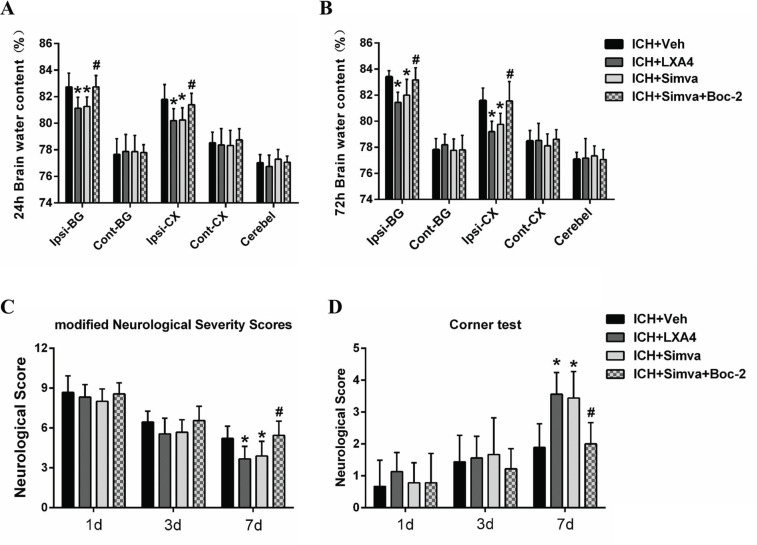
**The protective effect of simvastatin on brain edema and neurological deficits was abolished by Boc-2.** (**A** and **B**). The brain water content in both the ipsilateral basal ganglia and cortex of the LAX4 group and Simva. group was much lower than those in the Veh-group at 24 h and 72 h after ICH, but Boc-2 weakened the anti-edema effect of simvastatin on ICH. Values are presented as the means±SD, n=6 rats per group; *P<0.05 compared to the Veh group; #*P<*0.05 compared to the Simva group. Ipsi-BG, ipsilateral basal ganglia; Con-BG, contralateral basal ganglia; Ipsi-CX, ipsilateral cerebral cortex; Con-CX, contralateral cerebral cortex; Cerebel, cerebellum. (**C** and **D**) ICH rats that received exogenous LXA4 and simvastatin treatment presented a lower neurological score on the mNSS and a higher score for the corner test on Day 7 after ICH. Similarly, the FPR2 antagonist Boc-2 reversed the neuroprotection of simvastatin on ICH. Values are presented as the means±SD, n=6 rats per group; *P<0.05 compared to the Veh group; #*P<*0.05 compared to the Simva group.

We measured the brain water content of animals in each group and calculated their neurological scores to further investigate the role of FPR2 in simvastatin-mediated protective effects on the brain after ICH. The brain water contents in the ipsilateral basal ganglia and cortex of the LAX4 group were reduced compared with those in the Veh-group at 24 h and 72 h after ICH (Figs. **5A** and **B**). Notably, animal models treated with simvastatin showed a trend similar to that of the LXA4 group, but the anti-edema effect of simvastatin on ICH was weakened by Boc-2, a selective FPR2 antagonist (Figs. **5A** and **B**). Furthermore, as shown in Figs. (**5C** and **D**), ICH rats that received exogenous LXA4 treatment (as an FPR2 agonist) presented a lower neurological score on the mNSS and a higher score for the corner test on Day 7 after ICH.

### 3.6 Simvastatin Exerted a Pro-apoptotic Effect on PMNs in the Acute Stage after ICH *Via* the LXA4/FPR2/p38 MAPK Signaling Pathway

**Fig. (6):**
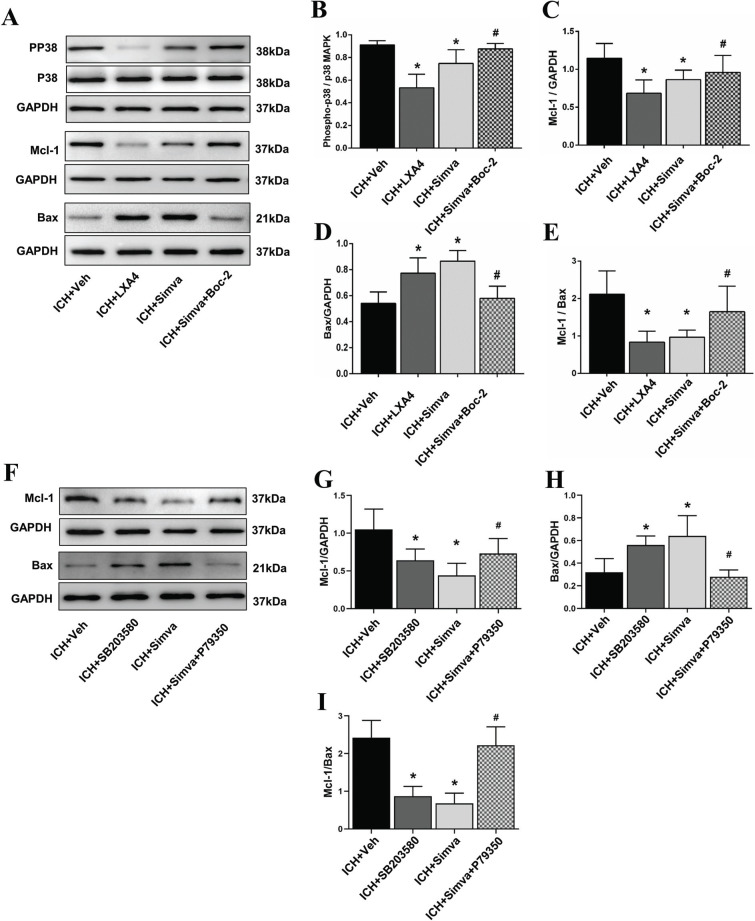
**The FPR2/P38 MAPK signaling pathway plays a key role in simvastatin-induced PMN apoptosis in the acute stage after ICH.** We explored the downstream signal pathway of FPR2 in the isolated peripheral PMNs at 24 h post-ICH. (**A-E**) Exogenous LXA4 and simvastatin decreased phosphorylation p38 and anti-apoptotic protein Mcl-1 levels while increasing pro-apoptotic protein Bax levels. Thus, the lower Mcl-1 to Bax ratio (a decreased ratio represents the induction of apoptosis) in both LXA4 and Simva-groups may explain the aforementioned PMN apoptosis. Furthermore, all the above effects induced by simvastatin were reversed by Boc-2. (**F-I**) Simvastatin led to a similar Mcl-1/Bax ratio reduction as SB203580 (a p38 MAPK inhibitor), but it was abolished by P79350 (a p38 MAPK agonist). These findings suggest that P38 MAPK may be an important downstream pathway of simvastatin-mediated neutrophil apoptosis in ICH rats. Values are presented as the means±SD, n=3 rats per group; *P<0.05 with the Veh group; #*P<*0.05 with the Simva group.

**Fig. (7):**
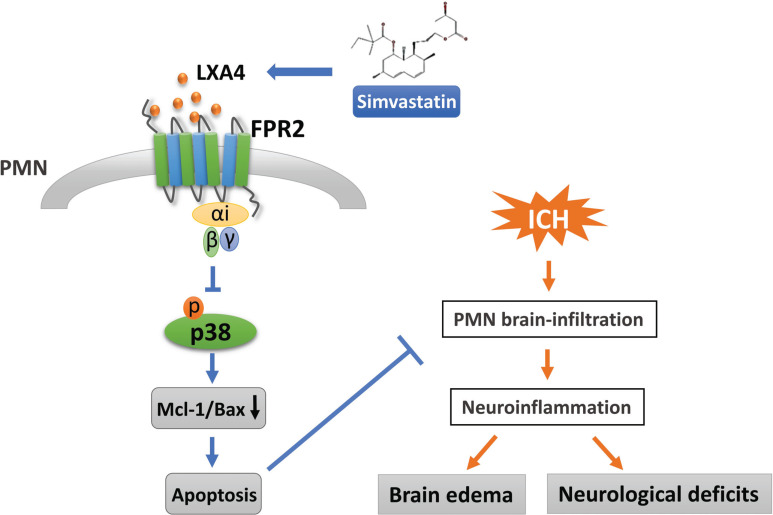
**Schematic of the proposed mechanisms.** Simvastatin-triggered LXA4 level up-regulation selectively activated the G protein-coupled receptor FPR2 on peripheral PMNs in the early stage of ICH, through which simvastatin inhibited the phosphorylation of P38 and decreased the Mcl-1/Bax ratio, which then accelerated the apoptosis of circulating PMNs and decreased PMN infiltration into the brain, and finally attenuated ICH-mediated neuroinflammation and brain injury.

We treated the animals with simvastatin, the FPR2 agonist LXA4, the FPR2 antagonist Boc-2, the p38 MAPK inhibitor SB203580, and the p38 MAPK agonist P79350 and analyzed the levels of downstream proteins to elucidate the downstream signaling pathway responsible for FPR2-mediated PMN apoptosis. Twenty-four hours after ICH, PMNs were isolated from the circulating blood of rats and then detected using western blot analysis. As shown in Figs. (**6A** and **B**) the FPR2 agonist LXA4 markedly reduced the level of phosphorylated p38 compared with that in the control group. In addition, the expression of the anti-apoptotic protein Mcl-1 was markedly inhibited by LXA4 (Fig. **6C**), while the level of the pro-apoptotic protein Bax was significantly upregulated (Figs. **6D** and **E**). Rats treated with LXA4 displayed a lower Mcl-1 to Bax ratio (the Mcl-1/Bax ratio plays a key role in modulating neutrophil survival), resulting in more PMN apoptosis than the Veh group. Notably, the Simva-group exhibited a similar trend of pp38/Mcl-1/Bax levels to the LXA4 group. However, co-treatment with Boc-2 abrogated the pro-apoptotic effect of simvastatin on PMNs following ICH. Next, we further assessed the relationship between the p38 MAPK signaling pathway and PMN apoptosis. As displayed in Figs. (**6F**-**I**), SB203580 (p38 MAPK inhibitor) dramatically reduced Mcl-1 expression in PMNs while increasing Bax levels after ICH, which led to a lower Mcl-1/Bax ratio. Notably, ICH rats treated with simvastatin presented a similar change in the Mcl-1/Bax balance as SB203580-treated rats, but the p38 MAPK agonist P79350 abolished this effect (Fig. **7**).

## 4 DISCUSSION

In this study, we investigated the potential mechanism of simvastatin-induced PMN apoptosis in a rat model of ICH. The major findings of this study are described below. (1) Simvastatin significantly elevated the plasma level of LXA4 (an endogenous FPR2 agonist) after ICH. (2) Similar to simvastatin, exogenous LXA4 effectively promoted circulating PMN apoptosis in the early stage of ICH and restricted the secondary brain injury triggered by PMNs invading the brain. (3) Boc-2, a selective antagonist of FPR2, dramatically abolished the neuroprotective effects of simvastatin on ICH. (4) The results of further experiments suggest that the FPR2/p38 MAPK signaling pathway plays an important role in simvastatin-induced PMN apoptosis.

Statins are associated with improved outcomes in patients with ischemic stroke [27]. In addition to their most familiar effect of reducing cholesterol levels, statins also exert pleiotropic effects, such as anti-inflammatory activity, promoting neurogenesis and protecting the blood-brain barrier [28, 29]. Thus, statins are attractive candidates for developing a neuroprotective strategy. Over the years, a series of retrospective cohort studies have reported that inpatient statin use is associated with improved outcomes following acute ICH [6-10]. Recently, accumulating evidence supports the hypothesis that a higher PMN count and neutrophil-to-lymphocyte ratio (NLR) in the early stage of ICH leads to a poorer outcome [11-14]. Thus, in a proof-of-concept experiment using a rat model of ICH, we found that simvastatin effectively decreased the peripheral PMN count, NLR and neutrophil brain invasion and attenuated brain edema and neurological deficits following ICH [15]. However, to date, the related molecular mechanisms by which simvastatin regulates PMN apoptosis are still unclear.

Lipoxins (LXs), which are lipids derived from arachidonic acid, exert anti-inflammatory effects by catalyzing arachidonic acid degradation by different lipoxygenases through a cross-cellular pathway [30]. Several recent studies have reported that LXA4 exerts a protective effect on CNS injuries, such as subarachnoid hemorrhage (SAH) and ischemic stroke [18, 19]. According to previous studies, LXA4 reduces PMN infiltration in SAH rats by activating FPR2 [18], consistent with the results of our research. However, the author only focused on the effect of LXA4 on microglial FPR2 and did not explore the effect and mechanism of LXA4 on FPR2 expressed on the PMN surface. More recently, contrary to our results, Futokoro and his colleagues reported that BML-111 (an agonist of FPR2) did not show a significant reduction in the number of perihematomal neutrophils [17]. The different results may be attributed to the different ICH models and FPR2 agonists. In their study, Futokoro *et al.* adopted the mouse model that received collagenase injection, while we employed the rat model with autologous blood injection. González-Herrera *et al.* [21] found that simvastatin assists cyclooxygenase-2 (COX-2) in converting arachidonic acid to LXA4 and exerts anti-inflammatory effects. Consistent with these findings, in the present study, simvastatin significantly increased the plasma level of LXA4 (an endogenous FPR2 agonist) at 24 h after ICH. Similar to simvastatin, exogenous LXA4 effectively promoted circulating PMN apoptosis in the early stage of ICH and restricted the secondary brain injury triggered by PMNs that infiltrated the brain.

In recent years, LXA4 has been reported to exert a noticeable anti-inflammatory and neuroprotective effect by reducing the activity of p38 MAPK through a mechanism mediated by FPR2 [18, 20, 31]. In the present study, we found that Boc-2, a selective antagonist of FPR2, dramatically abolished the neuroprotective effects of simvastatin on ICH. In addition, the FPR2 agonist LXA4 markedly reduced the level of phosphorylated p38. Moreover, simvastatin induced a similar reduction in the Mcl-1/Bax ratio to SB203580 (p38 MAPK inhibitor), but it was abolished by P79350 (p38 MAPK agonist). These data suggest that the FPR2/p38 MAPK signaling pathway plays an important role in simvastatin-induced neutrophil apoptosis. In addition to FPR2/p38 MAPK, many other LXA4-targeted downstream signaling pathways have been reported in some newly published preclinical studies [32-35]. Li *et al.* [32] found that LXA4 exerted protective effects on focal cerebral ischemia-reperfusion injury by regulating microglia polarization through the Notch signaling pathway. LXA4 decreased the expression of Notch-1, Hes1, iNOS and CD32, which are associated with M1 microglia differentiation. In contrast, LXA4 upregulated the expression of Hes5, Arg-1 and CD206, driving the M2 phenotype in microglia. Furthermore, DAPT, a Notch signaling pathway inhibitor, significantly mitigated the effect of LXA4 on microglia differentiation. In another study, LXA4 also suppressed Erastin-induced ferroptosis of spinal cord neurons *in vitro via* stimulating the AKT/Nrf2/HO-1 signaling pathway [34]. Furthermore, Li *et al.* [33] discovered that LXA4 effectively attenuated paraquat-induced acute lung injury in an experimental rat model, possibly by down-regulating inflammation-related signaling molecules such as toll-like receptor 4 (TLR4), myeloid differentiation primary response 88 (Myd88), nuclear factor (NF)B p65, phosphoinositide 3kinase (PI3K), and pAKT.

There are a few limitations to this study. First, statins and clinical outcomes in ICH patients are controversial. The primary and secondary prevention of ischemic stroke has been proven with statin use. However, there was some concern about a link between a high dose of statin and ICH and the risk of new-onset diabetes mellitus [36, 37]. Future experimental studies with a rat model would be valuable to understanding simvastatin's effect in ICH. Additionally, there remains an urgent need to find compounds for neuroprotection or nerve recovery during or after stroke. Increasing network pharmacology studies suggest that active ingredients from traditional Chinese medicines may be a potential candidate for anti-inflammation treatment for ICH [38, 39]. Second, we focused on p38 MAPK in this study. Recent data has indicated that other signaling pathways may also be involved in the effect of LXA4 on ICH [32-35]. Whether and how Notch, TLR4 and AKT also play a role as the downstream factors of FPR2 signaling in the context of ICH remains to be investigated in future studies. Third, we focused on the LXA4 level detection in the peripheral plasm but missed the LXA4 expression changes in brain tissue. Since a large number of FPR2 receptors are also expressed on the surface of microglia [18], it is not clear whether simvastatin or exogenous LXA4 also causes microglia-mediated inflammatory regulation, and further experimental confirmation is needed. Moreover, it would still be critical to design an *in vitro* study to further elucidate the interaction effect and correlational pathway between LXA4 and neutrophil apoptosis.

## CONCLUSION

The present study demonstrated that simvastatin might activate the FPR2 receptor on peripheral neutrophils *via* up-regulating the endogenous ligand LXA4, thus exerting pro-apoptosis and anti-inflammatory effects on a rat model of ICH through the FPR2/p38 MAPK signaling pathway. Therefore, simvastatin or LXA4 may be a promising and safe therapeutic option for ICH. Large animal experiments and clinical trials are required to further explore the efficacy and safety of future studies.

## Figures and Tables

**Fig. (1):**
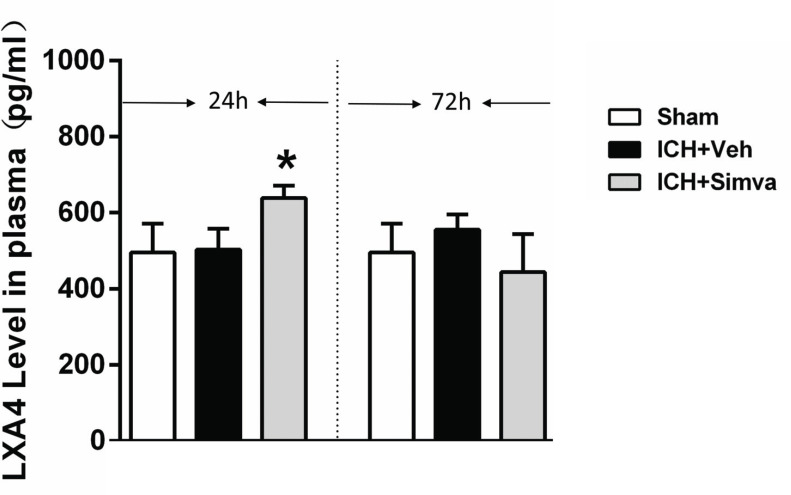
**Simvastatin significantly increased the plasm level of LXA4 (endogenous agonist for FPR2) at 24 h after ICH.** An ELISA test was performed to determine the plasma LXA4 level at 24 h and 72 h after ICH to investigate the relationship between simvastatin and LXA4 in rats with ICH. Compared with the sham and control groups, the ICH+Simva group presented higher levels of LXA4 at 24 h but no significant difference at 72 h. These data indicate that simvastatin upregulated pro-resolving mediator LXA4 levels in the early stage of ICH. Values are presented as means±SD, n=4 per group; *P<0.05 ICH-Simva versus ICH-Veh group. ICH, intracerebral hemorrhage; Simva, simvastatin; Veh, vehicle.

**Table 1:** Detailed information about antibodies.

**Antibody**	**Manufacturer**	**Concentration**
Rabbit anti-rat Phospho-p38 MAPK	Cell Signaling, USA	1:1000
Rabbit anti-rat p38 MAPK	Cell Signaling, USA	1:1000
Rabbit anti-rat MCL-1	Cell Signaling, USA	1:1000
Mouse anti-rat Bax	Abcam, USA	1:500
Rabbit anti-rat TNF-α	Affinity Biosciences, USA	1:500
Mouse anti-rat IL-6	Santa Cruz Biotechnology, USA	1:100
Rabbit anti-rat C3	Abcam, USA	1:1000
Mouse anti-rat α-Tubulin	Boster Biological Technology, China	1:1000
Rabbit anti-rat GAPDH	bs-2188R; BIOSS, Beijing, China	1:1000
Goat anti-rabbit IgG-HRP	ZSGB-BIO, China	1:2500
Goat anti-mouse IgG-HRP	ZSGB-BIO, China	1:2500
Rabbit anti-rat Myeloperoxidase	Abcam, USA	1:100
Goat anti-rabbit IgG 555	Beyotime, China	1:300

## Data Availability

The data supporting this study's findings are available from the corresponding author [JS] upon reasonable request.

## LIST OF ABBREVIATIONS

COX-2: Cyclooxygenase-2
FPR2: Formyl Peptide Receptor 2
ICH: Intracerebral Hemorrhage
LXA4: Lipoxin A4
MAPK: Mitogen-Activated Protein Kinase
Mcl-1: Myeloid Cell Leukemia 1
MPO: Myeloperoxidase
PI: Propidium Iodide
PMN: Polymorphonuclear Neutrophil
SAH: Subarachnoid Hemorrhage
